# Value chain hygiene practices and microbial contamination of street and market vended ready-to-eat grasshopper, *Ruspolia differens* in Uganda: Implications for food safety and public health

**DOI:** 10.1016/j.heliyon.2024.e25614

**Published:** 2024-02-08

**Authors:** Karlmax Rutaro, Joseph Hawumba, Jane Nakimuli, Julius Mulindwa, Geoffrey M. Malinga, Rhona Baingana

**Affiliations:** a Department of Biochemistry & Sports Science, School of Biological Sciences, College of Natural Sciences, Makerere University, P.O Box 7062, Kampala, Uganda; b Laboratory of Microbiology, Department of Biochemistry & Sports Science, School of Biological Sciences, College of Natural Sciences, Makerere University, P.O Box 7062, Kampala, Uganda; c Department of Biology, Gulu University, P.O Box 166, Gulu, Uganda

**Keywords:** Street foods, Edible grasshopper, Edible bush cricket, Sanitation

## Abstract

Food safety is a major public health issue particularly in developing countries. Ready-to-eat street-vended foods contribute significantly to dietary intake in urban and peri-urban areas, but with elevated public health risk. In this study, hygiene and food safety practices as well as the microbial contamination in Uganda's edible grasshopper value chain were evaluated."A total of 29 grasshopper-processing households participated, and grasshopper samples collected. Indicator pathogens were analyzed using standard microbiological methods. In Kampala 50% and in Masaka 12% households had earth floors. All households in Kampala were one or two-roomed dwellings with no separate room as a kitchen, and shared a toilet. In contrast, 59% of households in Masaka had three or more rooms, 35% had a separate room for a kitchen and 47% did not share a toilet. 83% households in Kampala and 56% in Masaka obtained drinking water from public taps. Handwashing was inadequate and none of the actors was observed to wash their hands after taking a break or handling waste. For vendors, wearing protective clothing was not common, with only 28.5% in Kampala and 30.8% in Masaka wearing an apron. Containers for vending grasshoppers were largely uncovered and the utensils for measuring the grasshoppers were left mainly uncovered. Indicator organisms, *Escherichia coli* and *Salmonella typhimurium,* were detected. *E. coli* was the most common contaminant, but with lower levels in Masaka compared to Kampala. *S. typhimurium* was mainly a burden in Kampala. Our findings demonstrate that there are enormous contributors to poor hygiene and sanitation along the edible grasshopper value chain. The existence of pathogenic bacteria such as *E. coli* in ready-to-eat foods imply that their consumption poses a health risk.

## 1 Introduction

Street foods are ready-to-eat (RTE) foods and beverages, processed or fresh that are sold at stationary locations or by mobile vendors in streets and open places as opposed to stores and licensed establishments [1]. These foods contribute significantly to the diet of many, and their consumption is an essential contributor to the dietary intake of mostly urban and peri-urban populations in developing countries [2]. Street foods are responsible for daily energy intake ranging from 13 to 50% in both adults and children [2]. Street foods present several advantages to the urban poor as they are convenient, cheap, and easily accessible and a source of income for many who otherwise would not have livelihoods. Unfortunately, the street food trade in many countries is largely unregulated, associated with poor hygiene and safety issues, and is thus considered a public health threat [3].

Concerns related to water and sanitation facilities, as well as the knowledge and expertise of street food handlers are rising [4]. For example, in an urban area in Brazil, 95% of street vendors did not wash their hands between food and money transactions and rest room breaks, and 100% did not have access to a water supply [5]. Relatedly, a study conducted in Cape Town, South Africa, showed that 77% of vendors handled food and money simultaneously, and soap was not available at 86% of stalls [6]. Literature, mainly from Africa, indicates that major life-threatening pathogens such as *Escherichia coli, Salmonella typhimurium* and *Staphylococcus aureus* are prevalent in street foods [7]. A study in Uganda showed that *S. aureus* was present in about 85% of beef and chicken samples and in 75% of goat meat samples, while *E. coli* was present in up to 50% of meat samples hawked at markets along highways [8]. Similarly, a study by Mugampoza et al. [9] found *E. coli* levels in 100% and 60%, respectively of all studied street foods sold in Nakawa and Naguru Parishes within Kampala City.

Edible insects are a potential food security solution because of their nutritive value and low environmental impact [10]. However, the presence of pathogenic microorganisms commonly associated with edible insects and their products is of great concern [11]. This issue could be related to how insects are handled, processed and/or packaged [12]. A review of articles published between 2000 and 2019 on a variety of edible insects, both raw and cooked, showed complex ecosystems, with marked variations in microbial load and diversity including mesophilic aerobes, bacterial endospores, *Enterobacteriaceae*, lactic acid bacteria, psychrotrophic aerobes, and fungi, and potentially harmful species (i.e., pathogenic, mycotoxigenic, and spoilage microbes) [12].

In Uganda, the long-horned edible grasshopper, *Ruspolia differens* (Serville) (Orthoptera: Tettigoniidae), locally known as ‘nsenene’ is a common street-vended food and is considered a delicacy [13]. During the swarming seasons of April–May and November–December each year [14], they are hawked on the streets of major cities and towns around the Lake Victoria basin, including Kampala and Masaka Cities. Prior to hawking on streets, primary “processors”, usually low-income slum dwellers, pluck off the appendages (wings and legs), and at times fry the grasshoppers ahead of distribution to market vendors and hawkers who sell them, raw or fried, on the city streets and in the suburbs [14]. Previous studies of raw and fried edible grasshoppers in Uganda and Tanzania reported high microbial counts and potential pathogens, including *Campylobacter* and *Staphylococcus* [15,16], making edible grasshoppers a potential source of foodborne diseases. Despite its increased recognition as a food resource and a major protein source [10], the edible insect value chain still has policy loopholes regarding harvesting, transportation, trading and processing [17], leading to increased risk of contamination [3], thus creating opportunity for disease outbreaks caused by the microbial and chemical food contaminants. For public health purposes, it is therefore important to understand how each actor in the edible grasshopper value chain potentially contributes to the contamination of processed grasshoppers, especially those marketed as RTE. However, the information on the microbial safety and quality of RTE street-vended edible insect products sold in Ugandan cities is limited. Therefore, the present study was designed to document and evaluate the hygiene practices of key actors in the value chain of RTE edible grasshoppers in the cities of Kampala and Masaka. Specifically, we set out to: (i) document the hygiene practices along the RTE edible grasshopper value chain, including the personnel involved and (ii) determine the level of microbial contamination in the RTE grasshoppers at different stages of the value chain. The findings of this study have the potential to inform public health policy, and to strengthen regulation of market- and street-vended RTE edible grasshoppers as well as other street-vended foods.

## 2 Materials and methods

### 2.1 Study site

**Fig. 1:**
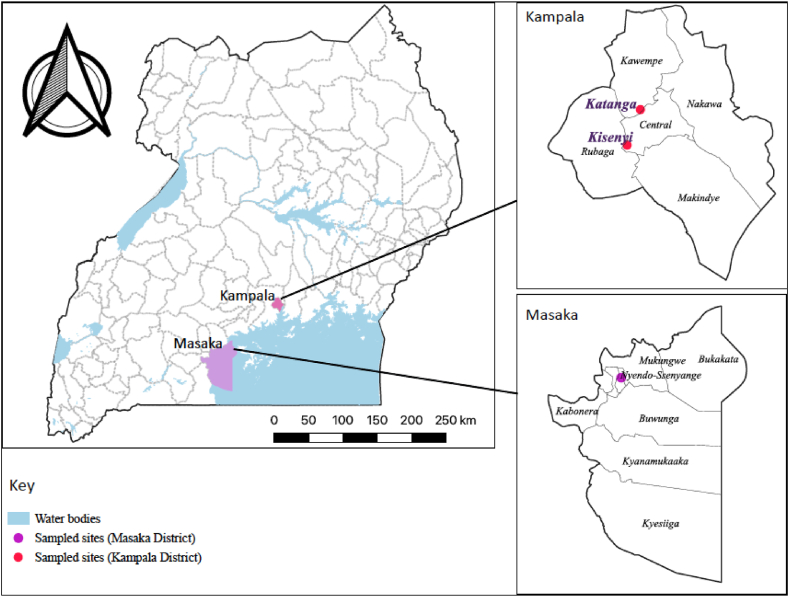
Map showing the location of the study areas in Kampala and Masaka districts, Uganda. The base map (shapefile) highlighting the country and district boundaries was obtained from UBOS [19].

The study was carried out in the cities of Kampala and Masaka located in central Uganda (Fig. 1). These cities were purposively selected because both are areas where edible grasshoppers are in very high demand and are widely consumed. Masaka is considered the traditional source of edible grasshopper and is the leading supplier for Kampala City. Katanga and Kisenyi in Kampala city and Nyendo-Ssenyange in Masaka city were purposively selected because they have a high concentration of edible grasshopper businesses. In addition, Nyendo-Ssenyange is the main grasshopper-harvesting hub [18]. Coincidentally, these areas are also characterized largely by informal settlements (slums). Most dwellers are low-income earners who shift trade to grasshopper harvesting and processing during the main swarming seasons in April–May and November–December each year.

### 2.2 Study design and data collection tools

This was a cross-sectional mixed methods study with both qualitative and quantitative approaches. Data was collected by trained field assistants in November and December 2021 during the grasshopper harvest season. A questionnaire adapted from UBOS [20] was used to collect sociodemographic and household characteristics, while observation check lists adapted from Hill et al. [6] were used to collect food hygiene practices and sanitation status of the grasshopper value chain actors (vendors and processors). The questionnaire was pre-tested in Nateete, a city suburb to the west of Kampala, with similar social, economic and demographic dynamics to the sampled areas and appropriate modifications made. Questions asked included the socio-demographic characteristics of the household heads (e.g., age, native language and housing infrastructure, food handling practices and food safety knowledge of the vendors, and access to clean water supply and other sanitary facilities). The observation checklists assessed the hygienic practices of food handlers, waste handling and disposal, food handling practices and sanitary facilities, including toilet and hand washing facilities. The hygienic practices were evaluated as either yes or no, then expressed in proportions.

### 2.3 Participant enrolment

Participation in the study was open to all active actors (processors and vendors) in the selected areas. All enrolment procedures were conducted at the study site by the study team. Prior to participant enrolment, the study investigators held meetings with the area Local Councils and the leadership of the grasshopper actors, the “Basenene”-a derivation from the local name of the edible grasshopper, “nsenene”, in each study area to confirm their interest in the study, establish contact persons, and obtain the number of the actors in each locality. Given the informal nature of this trade, vendor and processor lists were not readily available with the area leadership nor the “Basenene”. Therefore, snowball sampling was used to locate all the vending and processor households.

### 2.4 Data collection

For Masaka, all HHs (17; M1- M17) involved in grasshopper processing were included in the study. However, for Kampala, due to the irregular grasshopper season in November–December 2021 and the intermittent supply of grasshoppers to Kampala [21], the selected study areas were visited till late December to identify, interview and survey all participating HHs (12; K1–K12). All HHs visited were interviewed and grasshoppers were sampled as follows: fresh, with wings and legs (Sample 1); “dressed” grasshoppers with wings and legs plucked off (Sample 2) and fried RTE grasshoppers (Sample 3). Then, individual hawkers/vendors supplied with RTE grasshoppers from the interviewed households were followed and observed using a checklist between 12:00–14:00 h, the peak time for RTE grasshopper sales. Each vendor was closely observed for approximately 2 h to note behaviours before and during active trade. A sample (Sample 4) of RTE grasshoppers was then purchased from each of the hawkers/vendors who had been followed. Indicator microorganisms of poor hygiene, such as *E. coli* and *S. typhimurium* were tracked at each stage of the value-addition chain. For households K08 and K10, it was not possible to obtain samples for the final stage from the vending points (Sample 4). Street vendors are reported to be at risk of arrest by city authorities, who usually confiscate their merchandise [22]. To circumvent this, vendors opt to hawk the grasshoppers in bars at night. Households K09 and K11 in Kampala reported exporting their processed grasshoppers to the UK and USA, as reported also by Mmari et al. [23]. Therefore, we could only obtain relevant data up to stage 2 for these households. Households M13, M14 and M15 indicated a specialization in trading only in fresh grasshoppers and in “dressed” grasshoppers with wings and legs plucked off (stage 1 and 2 samples), and only fried the leftover unsold ones later in the night, for selling at a later date, ostensibly at a higher price. Therefore, the data collectors could only obtain samples 1, 2 and 4 from the indicated HHs. Households M16 and M17 reported removing appendages in the wee hours, thus the grasshoppers had already undergone the first stage of processing by the time the data collectors arrived at the HHs. All samples were immediately packaged into sterile Whirl-Pak bags (Nasco, USA) and placed in a cold box, with ice packs, maintained at approximately 5 °C. Samples from Kampala were immediately transported to the Microbiology Laboratory, Department of Biochemistry, Makerere University on the day of collection. Samples from Masaka, about 140 km away from Kampala, were frozen and later transported in a portable freezer to the same laboratory where they were transferred to −20 °C until analysis.

### 2.5 Assessment of microbial contamination along the value chain

Microbial assessment was carried out following standard procedures as outlined in [24], with minor modifications. Briefly, 10 g portions of each grasshopper sample were suspended in 100 mL of distilled water in conical flasks and agitated gently for 1 h to enable suspension of any microbes present. The suspension was decanted off, centrifuged (Hitachi Centrifuge, SCT 5 B, Hitach Koki Co. Ltd, Tokyo, Japan) at 5000 rpm for 10 min at room temperature, and the pellet stored at −80 °C. Aliquots of 1 mL of each sample were pipetted into sterile microfuge tubes and labelled according to the household number and sampling site for further analysis of culturable microorganisms. To determine the presence of potential pathogens in the samples, aliquots of 100 μL from each suspension were serially diluted up to 10^3^. Then, 100 μL of both 10^2^ and 10^3^ dilutions was plated onto MSA agar media (composed in g/L of mannitol [10 g], beef extract [1 g], tryptone [5 g], sodium chloride [75 g], phenol red [0.025 g] and Agar [15 g]) and XLD (HiMedia Laboratories Pvt. Ltd, Mumbai, India). Another aliquot of 100 μL was diluted to approximately 10 mL and filtered through a membrane filter (Pore size; 0.45 μm; 47 mm diameter; Advantec-Japan). Each filter was transferred to the *E. coli*-chromogenic medium (Condalab Conda S.A, Madrid, Spain). The plates were then incubated (SANYO Incubator, Sanyo Electric, Ltd, Japan) at 37 °C for 48 h. *E. coli* was identified by colonies turning blue on the *E. coli*-chromogenic medium, while presumptive *S. typhimurium* appeared red with a black center on XLD. Other colonies on XLD which also appeared red, possibly *Shigella* and other genera, did not have black centers and were grouped as other red colonies. It was also noted that various other types of bacteria were able to grow on XLD, producing yellow colonies. Additionally, various isolates were observed to grow at high salt concentrations and fermented mannitol, thus lowering the overall pH of the medium, signified by the phenol-red indicator turning yellow. Although MSA is primarily selective for *S. aureus*, other microbes were identified based on their colony morphologies, so the microorganisms that grew on MSA agar were generally grouped as mannitol fermenters. Representative isolates that could be cultured on both XLD and MSA were purified by repeated streaking on the same media and cultured in nutrient broth, then stored as glycerol stocks at −25 °C for further phylogenetic analyses.

### 2.6 Data analysis

Sociodemographic and household characteristics from the processor HHs and data from the observation checklists (HHs and street vendors) were entered into MS Excel, cleaned and then imported to Statistical Package for Social Scientists (SPSS version 23, SPSS Inc., Chicago, IL, USA) for analysis. Frequencies and proportions (%) of sociodemographic and household characteristics and checklist variables by study site were obtained. Hygiene criteria for minced meat [25], were used for the first and second processing stages (Samples 1 and 2, respectively), whereby the guideline lower limit for *E. coli* was 50 colony-forming units (cfu)/g and the upper limit 500 cfu/g. Uganda National Bureau of Standards (UNBS) Edible Insects Standard-2020 (US 2146:2020) [26], was used for evaluating the third processing stage (Sample 3) and for the vendors (Sample 4), whereby *E. coli, Staphylococcus* spp. and *Salmonella* spp. should be absent.

## 3 Results and discussion

### 3.1 Socio-demographic profile of the households

A total of 29 grasshopper-processing households participated in the study, i.e., 12 in Kampala and 17 in Masaka (Table 1). Majority of respondents were females (67% in Kampala and 53% in Masaka) and were household heads (83% in Kampala and 71% in Masaka), implying that most of the HHs involved in the grasshopper processing are female-headed, though a study by Odongo et al. [14] found 86% and 14% male and female, respectively, in grasshopper business in Uganda. However, Isingoma & Kwesiga [27] also found that 64% of HHs were female-headed in Banda, a low-income, densely populated (slum) suburb of Kampala City. Notably, while Kampala and Masaka are in the Central Region of Uganda with Luganda as the native language, none of the respondents in Kampala reported Luganda as their native language, compared to 14/17 (82%) of the respondents in Masaka. This indicates that the participant HHs in Kampala originated from outside Buganda, which is typical of Kampala's low-income, densely populated informal settlements (slums) where impoverished migrants from rural areas first settle. Indeed, a study exploring the livelihoods of the urban poor found that only 14% of respondents were born in Kampala [28]. Compared to Masaka, a higher proportion of participating households in Kampala had earth or sand floors (50% vs 12%); were one or two-roomed dwellings (100% vs 41%); no room dedicated as a kitchen (100% vs 65%) and shared a toilet (100% vs 53%) further reflecting the lower income status of the participating Kampala households. A study in Kampala by Mukiibi [29], showed that increased housing demand, rising land prices and growing urban poverty in the city had reduced low-income earners' accessibility to decent shelter. Most households obtained drinking water from public taps/standing pipes (83% in Kampala and 56% in Masaka), as also highlighted by Ssemugabo et al. [30]. However, in Masaka, some HHs also obtained their drinking water from public boreholes (11%) and protected wells/springs (6%).

**Table 1:** Sociodemographic and household characteristics of edible grasshopper processors in Kampala and Masaka cities, central Uganda.

Variable		Kampala (12)	Masaka (17)	All (29)
Age	Mean (SD)	37.2	34.8	39.0
	Min, Max	25, 49	17, 66	17, 66
Respondent	Females, number (%)	8 (67)	9 (53)	17 (59)
	Head of household	10 (83.3)	12 (70.6)	22 (75.9)
	Spouse	2 (16.7)	3 (17.6)	5 (17.2)
	Child	0	2 (11.8)	2 (6.9)
Native language	Runyakitara	11 (91.7)	3 (17.6)	14 (48.3)
	Lugbara	1 (8.3)	0	1 (3.4)
	Luganda	0	14 (82.4)	14 (48.3)
HH members	1,2	3 (25)	3 (17.6)	6 (21.7)
	3 to 5	7 (58.3)	4 (23.5)	11 (37.9)
	6 plus	2 (16.7)	10 (58.8)	12 (41.4)
Roof	Iron sheets	12 (100)	13 (76.5)	25 (89.3)
	Cement slab	0	3 (17.6)	3 (10.7)
	Other	0	1 (5.9)	1 (3.6)
Walls	Unburnt clay bricks with cement plastering	1 (8.3)	1 (5.9)	2 (6.9)
	Cement blocks	3 (25)	0	3 (10.3)
	Other	8 (66.7)	16 (94.1)	24 (82.8)
Floor	Earth or sand	6 (50)	2 (11.8)	8 (27.6)
	Cement	6 (50)	13 (76.5)	19 (65.5)
	Bricks	0	1 (5.9)	1 (3.4)
	Other	0	1 (5.9)	1 (3.4)
Rooms	1, 2	12 (100)	7 (41.1)	19 (65.5)
	3 plus	0	10 (58.8)	10 (34.5)
Bedrooms	1	12 (100)	6 (35.2)	18 (62.1)
	2 plus	0	11 (64.7)	11 (37.9)
Kitchen	Yes	0	6 (35.3)	6 (20.7)
	No	12 (100)	11 (64.7)	23 (79.3)
Lighting	Electricity National Grid	11 (91.7)	15 (88.2)	26 (89.7)
	Electricity Solar	0	2 (11.8)	2 (6.9)
	Other	1 (8.3)	0	1 (3.4)
Cooking	Charcoal	12 (100)	16 (94.1)	28 (96.6)
	Firewood	0	1 (5.9)	1 (3.4)
Drinking water^a^	Piped into dwelling	0	2 (11.1)	2 (6.7)
	Piped to yard/plot	1 (8.3)	1 (5.6)	2 (6.7)
	Public tap/standing pipe	10 (83.3)	10 (55.6)	20 (66.7)
	Bottled water	1 (8.3)	1 (5.6)	2 (6.7)
	Borehole in yard/plot	0	1 (5.6)	1 (3.3)
	Public borehole	0	2 (11.1)	2 (6.7)
	Protected public well/spring	0	1 (5.6)	1 (3.3)
Toilet^b^	Pit latrine - Covered - No slab	1 (8.3)	5 (27.8)	6 (20.0)
	Pit latrine - Covered - with slab	1 (8.3)	12 (66.7)	13 (43.3)
	Flush or pour flush toilet	10 (83.3)	1 (5.6)	11 (36.7)
Toilet shared	Yes	12 (100)	9 (52.9)	21 (72.4)
	No	0	8 (47.1)	8 (27.6)
Toilet handwash facility	Yes	8 (66.7)	15 (88.2)	23 (79.3)
	No	4 (33.3)	2 (11.8)	6 (20.7)
Handwash place observed	No specific place for handwashing	11 (91.7)	17 (100)	28 (96.6)
	Handwashing place observed	1 (8.3)	0	1 (3.4)

a Total for Masaka is 18 because one HH has two sources.

b Total for Masaka is 18 because one HH uses two types of toilet.

### 3.2 Hygiene and food safety practices of street food vendors and processors

Table 2A–C provide data on the hygiene and food safety practices of the grasshopper processing households and of the street vendors. Overall, at the HH level, handwashing was inadequate: only five out of all 29 participating HHs were observed to wash their hands after each processing stage; none were observed to wash their hands after taking a rest or handling waste; only five out of all 29 HHs had a handwashing facility with running water and only two out of all 29 HHs had a handwashing facility with soap. Regarding environment and equipment hygiene (Table 2B), raised racks for drying utensils were rare across both Kampala and Masaka (three out of all 29 HHs). Furthermore, four of the 12 HHs in Kampala and 12 of the 17 HHs in Masaka placed grasshoppers on the ground during the plucking of wings and legs (first stage of processing). Notably, compared to 10 out of 17 HHs in Masaka, only two of the 12 HHs in Kampala had a rubbish dump more than 15 m away from the processing area, characteristic of the low-income, densely populated informal settlements of Kampala. The impediments resulting in poor sanitation in urban communities in Uganda have previously been reported [30,31].

**Table 2:** Hygiene practices of edible grasshopper processors and vendors in Masaka and Kampala cities, Uganda.

A) Personal hygiene practices						
	Kampala (N = 12)		Masaka (N = 17)		All (N = 29)	
	Yes	No	Yes	No	Yes	No
**Part A: Personal Hygiene Practices**	n (%)	n (%)	n (%)	n (%)	n (%)	n (%)
Clothes are clean	10 (83.3)	2 (16.7)	13 (76.5)	4 (23.5)	23 (79.3)	6 (20.7)
Wearing a hairnet or head-cloth	3 (25.0)	9 (75.0)	4 (23.5)	13 (76.5)	7 (24.1)	22 (75.9)
Fingernails short and clean	10 (83.3)	2 (16.7)	13 (76.5)	4 (23.5)	23 (79.3)	6 (20.7)
Does not touch nose, mouth, hair, and skin	9 (75.0)	3 (25.0)	13 (76.5)	4 (23.5)	22 (75.9)	7 (24.1)
Does not lick fingers	10 (83.3)	2 (16.7)	13 (76.5)	4 (23.5)	23 (79.3)	6 (20.7)
Does not touch pimples or sores	10 (83.3)	2 (16.7)	14 (82.4)	3 (17.6)	24 (82.8)	5 (17.2)
Does not cough onto the grasshoppers	10 (83.3)	2 (16.7)	11 (64.7)	6 (35.3)	21 (72.4)	8 (27.6)
Not wearing rings on fingers	12 (100.0)	0 (0.0)	15 (88.2)	2 (11.8)	27 (93.1)	2 (6.9)
Does not have wounds or cuts on hands	12 (100.0)	0 (0.0)	13 (76.5)	4 (23.5)	25 (86.2)	4 (13.8)
Wound on the hand is covered with a plaster	NA	NA	NA	NA	NA	NA
Does not have flu-like symptoms	10 (88.9)	2 (11.1)	15 (88.2)	2 (11.8)	25 (86.2)	4 (13.8)
Hands washed after each processing stage	2 (16.7)	10 (83.3)	3 (17.6)	14 (82.4)	5 (17.24)	24 (82.8)
Hands washed after a break	0 (0.0)	12 (100.0)	0 (0.0)	17 (100.0)	0 (0.00)	29 (100.0)
Hands washed after handling waste	0 (0.0)	12 (100.0)	0 (0.0)	17 (100.0)	0 (0.00)	29 (100.0)
Hands washed after cleaning duties	0 (0.0)	12 (100.0)	3 (17.6)	14 (82.4)	3 (10.34)	26 (89.7)
Hand-wash place has running/flowing water	2 (16.7)	10 (83.3)	3 (17.6)	14 (82.4)	5 (17.24)	24 (82.8)
Hand-wash place has soap	0 (0.0)	9 (100.0)	2 (11.8)	15 (88.2)	2 (6.90)	24 (93.1)

B) Household Environment and Equipment Hygiene						
	Kampala (N = 12)		Masaka (N = 17)		All (N = 29)	
	Yes	No	Yes	No	Yes	No
**Processing area and equipment hygiene**	n (%)	n (%)	n (%)	n (%)	n (%)	n (%)
Utensils are clean	12 (100.0)	0 (0.0)	15 (88.2)	2 (11.8)	27 (91.7)	2 (8.3)
Wash place for utensils is clean	7 (58.3)	5 (41.7)	10 (58.8)	7 (41.2)	17 (58.3)	12 (41.7)
Drying rack for utensils available	2 (16.7)	10 (83.3)	1 (5.9)	16 (94.1)	3 (8.3)	26 (91.7)
Drying rack used to dry utensils	2 (16.7)	10 (83.3)	3 (17.6)	14 (82.4)	5 (16.7)	24 (83.3)
Grasshoppers are not placed on the ground	8 (66.7)	4 (33.3)	5 (29.4)	12 (70.6)	13 (45.8)	16 (54.2)
No domestic animals roaming around	9 (75.0)	3 (25.0)	13 (76.5)	2 (23.5)	22 (75.0)	7 (25.0)
No stagnant water nearby	10 (83.3)	2 (16.7)	17 (100.0)	0 (0.0)	27 (95.8)	2 (4.2)
Solid waste is safely disposed off	8 (66.7)	4 (33.3)	16 (94.1)	1 (5.9)	24 (75.0)	5 (25.0)
Wastewater is safely disposed off	9 (75.0)	3 (25.0)	12 (70.6)	5 (29.4)	21 (70.8)	8 (29.2)
Latrines are at least 15 m from the processing area	10 (83.3)	2 (16.7)	15 (88.2)	2 (11.8)	25 (87.5)	4 (12.5)
The rubbish dump is at least 15 m away	2 (16.7)	10 (83.3)	10 (58.8)	7 (41.2)	12 (37.5)	17 (62.5)
Not many flies hovering around (more than 10)	4 (33.3)	8 (66.7)	9 (52.9)	8 (47.1)	13 (41.7)	16 (58.3)
Fried grasshoppers are in a covered container	10 (83.3)	2 (16.7)	8 (47.1)	9 (52.9)	18 (58.3)	11 (41.7)
Rubbish e.g., left over food not scattered around	9 (75.0)	3 (25.0)	12 (70.6)	5 (29.4)	21 (70.8)	8 (29.2)
Fried grasshoppers are not put in dirty containers	9 (75.0)	3 (25.0)	13 (76.5)	4 (23.5)	22 (75.0)	7 (25.0)
Separate containers used (raw and fried)	9 (75.0)	3 (25.0)	16 (94.1)	1 (5.9)	25 (87.5)	4 (12.5)
Children are not playing around	4 (33.3)	8 (66.7)	14 (82.4)	3 (17.6)	18 (62.5)	11 (37.5)
Grasshoppers are washed before frying	12 (100.0)	0 (0.0)	17 (100.0)	0 (0.0)	29 (100.0)	0 (0.0)

C) RTE grasshopper vendors						
	Kampala (N = 7)		Masaka (N = 13)		All (N = 20)	
	Yes	No	Yes	No	Yes	No
RTE street food safety norms	n (%)	n (%)	n (%)	n (%)	n (%)	n (%)
Clothes are clean	5 (71.4)	2 (28.6)	13 (100.0)	0 (0.0)	18 (90.0)	2 (10.0)
Wearing apron or lesu or coat	2 (28.6)	5 (71.4)	4 (30.8)	9 (69.2)	6 (30.0)	14 (70.0)
Wearing a hairnet or head-cloth	1 (14.3)	6 (85.7)	1 (7.7)	12 (92.3)	2 (10.0)	18 (90.0)
Fingernails short and clean	5 (71.4)	2 (28.6)	12 (92.3)	1 (7.7)	17 (85.0)	3 (15.0)
Not touching nose, hair and skin during work	6 (85.7)	1 (14.3)	13 (100.0)	0 (0.0)	19 (95.0)	1 (5.0)
Does not lick fingers	7 (100.0)	0 (0.0)	12 (92.3)	1 (7.7)	19 (95.0)	1 (5.0)
Does not touch pimples or sores	7 (100.0)	0 (0.0)	13 (100.0)	0 (0.0)	20 (100.0)	0 (0.0)
Does not cough or sneeze	7 (100.0)	0 (0.0)	13 (100.0)	0 (0.0)	20 (100.0)	0 (0.0)
Does not have wounds or cuts on hands	7 (100.0)	0 (0.0)	13 (100.0)	0 (0.0)	20 (100.0)	0 (0.0)
Does not have flu-like symptoms	7 (100.0)	0 (0.0)	12 (92.3)	1 (7.7)	19 (95.0)	1 (5.0)
No stagnant water nearby	6 (85.7)	1 (14.3)	13 (100.0)	0 (0.0)	19 (95.0)	1 (5.0)
Latrine is at least 15 m away from vendor	7 (100.0)	0 (0.0)	11 (84.6)	2 (15.4)	18 (90.0)	2 (10.0)
Rubbish dump is at least 15 m from vendor	6 (85.7)	1 (14.3)	9 (69.2)	4 (30.8)	15 (75.0)	5 (25.0)
Not many flies (≥10) hovering around	6 (85.7)	1 (14.3)	10 (76.9)	3 (23.1)	16 (80.0)	4 (20.0)
Fried grasshoppers are in covered containers	5 (71.4)	2 (28.6)	5 (38.5)	8 (61.5)	10 (50.0)	10 (50.0)
No rubbish around the vending point	5 (71.4)	2 (28.6)	9 (69.2)	4 (30.8)	14 (70.0)	6 (30.0)
No bare hand touching the grasshoppers	7 (100.0)	0 (0.0)	11 (84.6)	2 (15.4)	18 (90.0)	2 (10.0)
Cover to grasshopper measuring utensils	3 (42.9)	4 (57.1)	2 (15.4)	11 (84.6)	5 (25.0)	15 (75.0)
Hand-washing point nearby	0 (0.0)	7 (100.0)	3 (23.1)	10 (76.9)	3 (15.0)	17 (85.0)

All HHs in Kampala and Masaka washed the grasshoppers before frying them. However, this may not necessarily have been motivated by hygiene and food safety, given that processors use ash or sand to counter the oiliness of the grasshoppers while plucking off the legs and wings. The main aim of washing the grasshoppers is to enhance the mouthfeel by removing the ash or sand. The use of protective clothing amongst vendors was not common: only two of the seven vendors observed in Kampala, and four out of 13 in Masaka wore an apron, while one in each of Kampala and Masaka wore a hairnet or head covering. Largely, RTE grasshoppers were not vended in closed containers (two out of seven vendors in Kampala and eight out of 13 vendors in Masaka). The utensils (spoon or cup) used to measure the grasshoppers were not covered for over half (four out of seven) of the vendors in Kampala and almost all (11 out of 13) vendors in Masaka. No vendors in Kampala had a hand-washing facility nearby, while only three out of 13 in Masaka did. Regardless of the country or product sold, African street vendors appear to share common unhygienic practices. For example, a study in Ethiopia [32] found that 88.6% of vendors did not wear aprons and 95% had their hair uncovered during cooking. Furthermore, all the vendors (100%) surveyed handled money with bare hands while serving food [32]. These drivers of poor hygiene were also observed in the current study (Table 2C).

### 3.3 Microbial contamination along the value chain

**Fig. 2:**
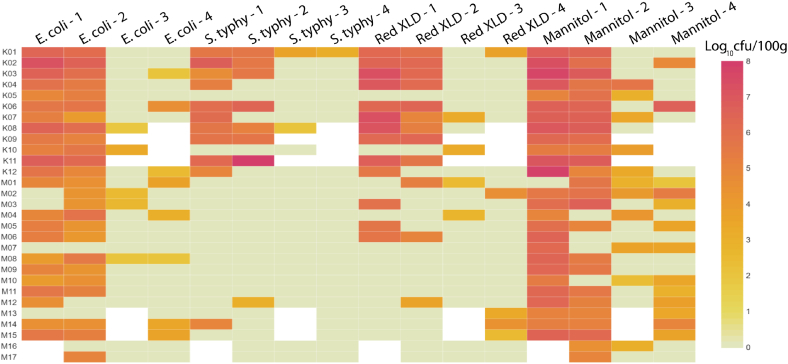
Heatmap showing indicator microorganisms (log cfu/100 g) at the different processing stages of the value chain. K01–K12 and M01-M17 represent grasshopper processing HHs in Kampala and Masaka, respectively. 1-unprocessed grasshoppers; 2-de-legged and de-winged; 3-fried at the HH; 4-vendor RTE grasshoppers. White cells: no samples. Mannitol: organisms able to grow on MSA medium and ferment mannitol; XLD: other red colonies able to grow on XLD, including *Shigella*. For households K08 and K10, it was not possible to obtain samples from the vending points. Households K09 and K11 reported exporting the grasshoppers. Households M13, M14 and M15 dealt in live grasshoppers, while for HHs M16 and M17, the grasshoppers had already undergone the first stage of processing by the time the data collectors arrived at the HHs.

A total of 105 edible grasshopper samples (27 sample 1, 29 sample 2, 24 sample 3 and 25 sample 4) were analyzed for the presence of bacterial pathogens. The results of microbial contamination along the grasshopper value chain are summarized in Fig. 2. The results revealed that in both Kampala and Masaka, unprocessed grasshoppers were contaminated with indicator microbes (Fig. 2). Overall, unprocessed grasshoppers were contaminated with *E. coli* (85.2%), *S. typhimurium* (40.7%), XLD fermenters (48.2%) and mannitol fermenters (96.3%). The presence of these indicator organisms in the raw, unprocessed grasshoppers suggests that some of the contamination may come from outside the value chain actors. Grasshoppers could potentially get contaminated from where they originate, given that they are thought to swarm to the point of harvest from “unknown sources”, although a study by Opoke et al. [33] suggests a local origin from which they aggregate and swarm upon maturity. Previous studies by Ssepuuya et al. [16] and Labu et al. [34] show that grasshoppers surveyed at the point of harvest already had high levels of contamination with potential human pathogens.

Contamination levels for all studied indicator organisms were reduced with processing (Fig. 2). This agrees with the findings of the study by Labu et al. [34], which observed that bacterial and fungal counts in processed grasshoppers were generally lower than in freshly harvested (unprocessed) raw grasshoppers. Heat processing, such as frying, would be expected to eliminate any contaminating microorganisms in the grasshoppers. Indeed, this was a general observation, except for the observed re-contamination in households K08 and K10 in Kampala and M02, M03 and M08 in Masaka (Fig. 2). Food re-contamination is largely associated with poor hygiene practices, such as not washing hands after handling waste or after taking a break [35]. In this study, handwashing was observed to be inadequate at the household level, with only five (17.2%) out of all 29 participating HHs in Kampala and Masaka observed to wash hands after each processing stage. This suggests that the actors were simultaneously handling both cooked and raw grasshoppers, and none washed their hands after taking a break or handling waste.

Contamination with *S. typhimurium* was mainly a burden among Kampala grasshopper processors compared to those in Masaka (Fig. 2). The major reservoirs of *Salmonella* are food animals, such as poultry, pigs and cows; however, humans, especially infected food handlers, and contaminated environments are also reservoirs of *Salmonella* [36]. Because the grasshoppers in this study were wild harvested, the presence of *S. typhimurium* suggests infected processors and/or contaminated environments in the Kampala processing HHs. This is supported by the HH characteristics data, as already discussed, exemplified by Kampala's low-income, densely populated informal settlements (slums).

It is also noteworthy that *S. aureus*, one of the key mannitol fermenters, is a usual constituent of the microbiota of the body, frequently found in the upper respiratory tract and on the skin in about 30% of humans [37]. However, it can also become an opportunistic pathogen and is a common cause of skin infections, respiratory infections, and food poisoning [38]. The presence of mannitol fermenters in the stage 3 (fried) and stage 4 (vended) grasshopper samples (Fig. 2) indicates that the RTE have potentially been contaminated by the food handlers themselves. Hand hygiene is key in the prevention of *Staphylococcus* infections, and RTE foods are especially risky if contaminated with *S. aureus*. As observed in this study, hand hygiene was poor, making RTE grasshoppers a hazard for *S. aureus* infection. *S. typhimurium*, *S. aureus* and other enteric pathogens associated with man are highly vulnerable to destruction by heat treatment and nearly all sanitizing agents. The fact that grasshoppers, like other RTE foods are usually consumed without further heating indicate that the consumers have a lot of confidence in the processors [39]. However, based on the findings of this study, this confidence is misplaced especially for the RTE grasshoppers vended in Kampala. As observed in this study (Table 2), access to clean water, good sanitation and proper waste management was inadequate in the surveyed areas, thus raising the possibility of transmission through contaminated water, utensils and environments during the preparation of RTE foods. The findings show that contamination and recontamination at all levels along the value chain is an evident risk for RTE grasshoppers in the study setting.

**Fig. 3:**
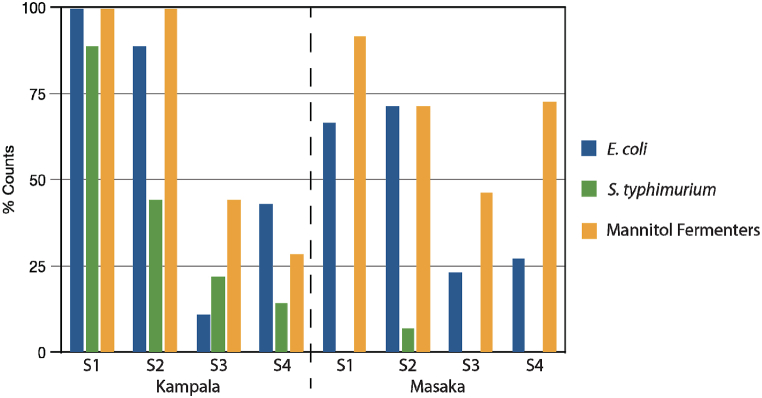
Percentage of households and vendors with grasshoppers categorized as hazardous by indicator organism along the value chain. Stage 1-unprocessed grasshoppers; 2-de-legged and de-winged; 3-fried grasshoppers at the HH; 4-vendor RTE grasshoppers. Masaka Stage 1 = 12 samples, Stage 2 = 14 samples, stage 3 = 13 samples; for Stages 1 and 2: Hazardous = >500 cfu/g. For Stages 3 and 4, hazardous = presence of the indicator organisms.

Generally, a lower proportion of samples from Masaka were contaminated by all classes of indicator organisms compared to those in Kampala (Fig. 3). The reason for this is unclear, but could be due to poor housing infrastructure and general poor sanitation in Kampala slums compared to Masaka, which had relatively better housing and sanitation infrastructure. A related study by Labu et al. [34] carried out in both localities found the mean bacterial counts in April–May (season 1) *grasshopper* samples from Masaka were significantly higher than those in samples from Kampala. However, there was no significant difference in November–December (season two) samples, although microbial species were most diverse in wild, freshly harvested samples.

## 4 Conclusion

Our findings demonstrate that there are enormous contributors to poor hygiene and sanitation along the value chain, resulting in possible contamination and re-contamination with potential pathogens. The existence of pathogenic bacteria such as *E. coli*, *S. typhimurium*, and *S. aureus* in RTE foods imply that consumption of these foods is a health risk for consumers. There is an urgent need for government and city public health departments to create awareness among street and market vendors and consumers through regular trainings on food safety and personal hygiene practices in food handling and inspection to prevent foodborne disease outbreaks. The street food hygiene and food safety norms detailed in the FAO Training Manual [40], if well utilized would facilitate RTE street food safety. Furthermore, since the grasshopper business generates income for participating households, it is recommended that Uganda National Bureau of Standards regulate the grasshopper value-chain business. Finally, government should provide basic social services/infrastructure, e.g., water, sanitation facilities to improve the working conditions of street vendors, including RTE grasshopper vendors.

## Ethics approval and consent to participate

All methods were performed in accordance with the relevant guidelines and regulations of the Declaration of Helsinki. The study protocol and informed consent documents were reviewed and approved by the Makerere University School of Social Sciences Research and Ethics Committee (No. MAKSSREC09.21.496) and registered with Uganda National Council for Science and Technology (No. HS1869ES). Participants provided written informed consent for their interviews.

## Funding statement

Authors acknowledge funding from the 10.13039/100004413International Foundation for Science (IFS), GRANT NO. I3-E−6584-1 to Karlmax Rutaro. The funding body had no role in the study design, data collection, analysis, and interpretation and in writing the manuscript.

## Data availability statement

All data generated or analyzed during this study are included in this manuscript.

## CRediT authorship contribution statement

**Karlmax Rutaro:** Conceptualization, Data curation, Formal analysis, Funding acquisition, Investigation, Methodology, Supervision, Writing – original draft, Writing – review & editing. **Joseph Hawumba:** Conceptualization, Formal analysis, Methodology, Writing – original draft, Writing – review & editing. **Jane Nakimuli:** Methodology, Writing – original draft, Writing – review & editing. **Julius Mulindwa:** Conceptualization, Formal analysis, Investigation, Methodology, Writing – original draft, Writing – review & editing. **Geoffrey M. Malinga:** Conceptualization, Formal analysis, Investigation, Methodology, Writing – original draft, Writing – review & editing. **Rhona Baingana:** Conceptualization, Data curation, Formal analysis, Investigation, Methodology, Software, Writing – original draft, Writing – review & editing.

## Declaration of competing interest

The authors declare that they have no known competing financial interests or personal relationships that could have an influence the work reported in this manuscript.
